# Development and validation of an artificial intelligence proof-of-concept tool for risk-based quality assessment of generic medicines: a South African case study

**DOI:** 10.3389/fmed.2026.1811333

**Published:** 2026-05-27

**Authors:** Lorraine Danks, Boitumelo Semete-Makokotlela, Christelna Reynecke, Seima Dijeng, Bathusi Kgosietsile, Japie van Tonder, Cornelia van Graan, Jason Hinch, Adriaan Kruger, Stuart Walker, Sam Salek

**Affiliations:** 1 School of Health, Medicine and Life Sciences, University of Hertfordshire, Hatfield, United Kingdom; 2 South African Health Products Regulatory Authority (SAHPRA), Pretoria, South Africa; 3 Botswana Medicines Regulatory Authority (BoMRA), Gaborone, Botswana; 4 Scigenix (Pty) Ltd., Pretoria, South Africa; 5 nuvoteQ (Pty) Ltd., Pretoria, South Africa

**Keywords:** artificial intelligence, BoMRA, generic medicines, Llama 3.1, low- and middle-income countries, risk-based assessment, SAHPRA, universal health coverage

## Abstract

**Introduction:**

Regulatory authorities in African countries face persistent challenges in ensuring the timely authorization of quality generic medicines due to limited technical capacity and extensive review timelines. The South African medicines regulator (SAHPRA) piloted a risk-based assessment (RBA) framework to expedite the Chemistry, Manufacturing, and Controls (CMC) evaluation of generic medicines. Building on this initiative, an artificial intelligence (AI)-driven tool, termed LEXI, was developed and validated to automate SAHPRA’s RBA triage for generic medicines and was externally validated at two African NRAs.

**Methods:**

LEXI was designed based on SAHPRA’s established RBA quality matrix, using 210 of its product dossiers, and was validated under the Good Automated Manufacturing Practice (GAMP5) framework. The system employed Retrieval-Augmented Generation (RAG) architecture with Meta’s Llama 3.1 large language model, supported by regulatory data integrations from the European Directorate for the Quality of Medicines (EDQM) and the World Health Organization Prequalification Programme. Validation comprised three qualification phases—installation (IQ), operational (OQ), and performance (PQ)—and was conducted across two African national regulatory authorities (NRAs): SAHPRA and the Botswana Medicines Regulatory Authority (BoMRA).

**Results:**

During external validation across 60 dossiers, LEXI demonstrated 91.7% overall predictive accuracy (55/60; 95% CI: 81.6-97.2%), with sensitivity of 96.9% (31/32; 95% CI: 83.8-99.9%) and specificity of 85.7% (24/28; 95% CI: 67.3-96.0%), with a median 91% time reduction; performance varied by criterion and dossier structure. Criterion-level accuracy exceeded 95% for most critical quality attributes (CQAs). Adaptations allowed for effective operation across different dossier structures and local regulatory environments, confirming its interoperability and robustness.

**Discussion:**

The validation of LEXI highlights the feasibility of secure, traceable AI integration into regulatory workflows. The tool augments human expertise by automating risk classification and dossier triage, enabling consistent, evidence-based decision support. These findings align with global regulatory trends at mature authorities, emphasizing AI’s role in improving regulatory agility while maintaining scientific rigor.

**Conclusion:**

LEXI represents a validated proof-of-concept for AI-assisted, risk-based regulatory assessment in resource-constrained settings. Its demonstrated efficiency and accuracy support its potential to enhance regulatory workflows and reduce review backlogs of generic medicines, pending further validation across a broader range of product types, including complex formulations, and diverse regulatory environments.

## 1 Introduction

National regulatory authorities (NRAs) in low- and middle-income countries (LMICs) in Africa face significant resource constraints and must, therefore, optimize the use of limited human and financial capacity when reviewing medicine applications (1, 2). Risk-based assessment (RBA) of the quality aspects of medicines has been gaining traction as this approach recommends focusing review on only the critical aspects of a product, thereby conserving resources and facilitating accelerated authorization without compromising patient safety. The World Health Organization (WHO)-Listed Authorities (WLAs) (3) and other regulatory institutions are increasingly implementing risk-based quality review practices, as advocated by the International Council for Harmonisation (ICH) of Technical Requirements for Pharmaceuticals for Human Use (4, 5), who defines quality risk management as “a systematic process for the assessment, control, communication and review of risks to the quality of the drug (medicinal) product across the product lifecycle” (4). One of these WLAs is the United States Food & Drug Administration (US FDA), who utilizes a Question-based Review (QbR) for the Chemistry, Manufacturing and Controls (CMC) or quality review of generic (multisource) medicine applications (6). The QbR guides reviewers in evaluating product quality and determining the risks related to manufacturing and design, and is focused on the critical quality (or pharmaceutical) attributes (CQAs) of a product throughout its assessment (6, 7). According to ICH Q8(R2), a “CQA is a physical, chemical, biological, or microbiological property or characteristic that should be within an appropriate limit, range, or distribution to ensure the desired product quality” (5). Examples of CQAs are purity, potency, dissolution rate, particle size, sterility (for sterile products), as well as pH and osmolarity (for injectables) and takes cognizance of the relevant dosage form (5). Through the QbR, more attention is paid to, for instance, complex dosage forms, narrow therapeutic index (NTI) active pharmaceutical ingredients (APIs) and high-risk manufacturing processes, with less significance attached to, and therefore less review of, lower-risk products such as immediate release solid oral products (6). According to the US FDA, this risk-based approach “maximizes economy of time, effort, and resources,” while preserving “the best practices of [the] current review system and organization” (6).

Given the socio-economic backdrop of African countries, the new registration application pipeline in these jurisdictions consists of more than 90% generic medicines (1, 2), with the CMC data of the majority of these products requiring full assessment, as documentation supporting reliance is oftentimes not available. Frequently, the API(s), dosage form(s) and manufacturing procedures are well known, having been previously evaluated by an NRA for similar generic products, with the knowledge embedded in the regulator’s institutional memory. Moreover, in many cases, either the API and/or the finished pharmaceutical product (FPP) has been previously reviewed and qualified by regulatory institutions, such as the European Directorate for the Quality of Medicines & Healthcare (EDQM) resulting in Certificates of Suitability (CEPs) (8), or Certificates of Prequalification (CPQs) (9), or are controlled via monographs contained in other reputable pharmacopoeias. Hence, a full review of all the aspects of generic product applications is neither practical, nor advisable, especially in view of the limited resources within African NRAs. To mitigate duplication of NRA efforts, a risk-based quality assessment approach for generic medicines, as has been instituted by the US FDA, is encouraged.

**FIGURE 1:**
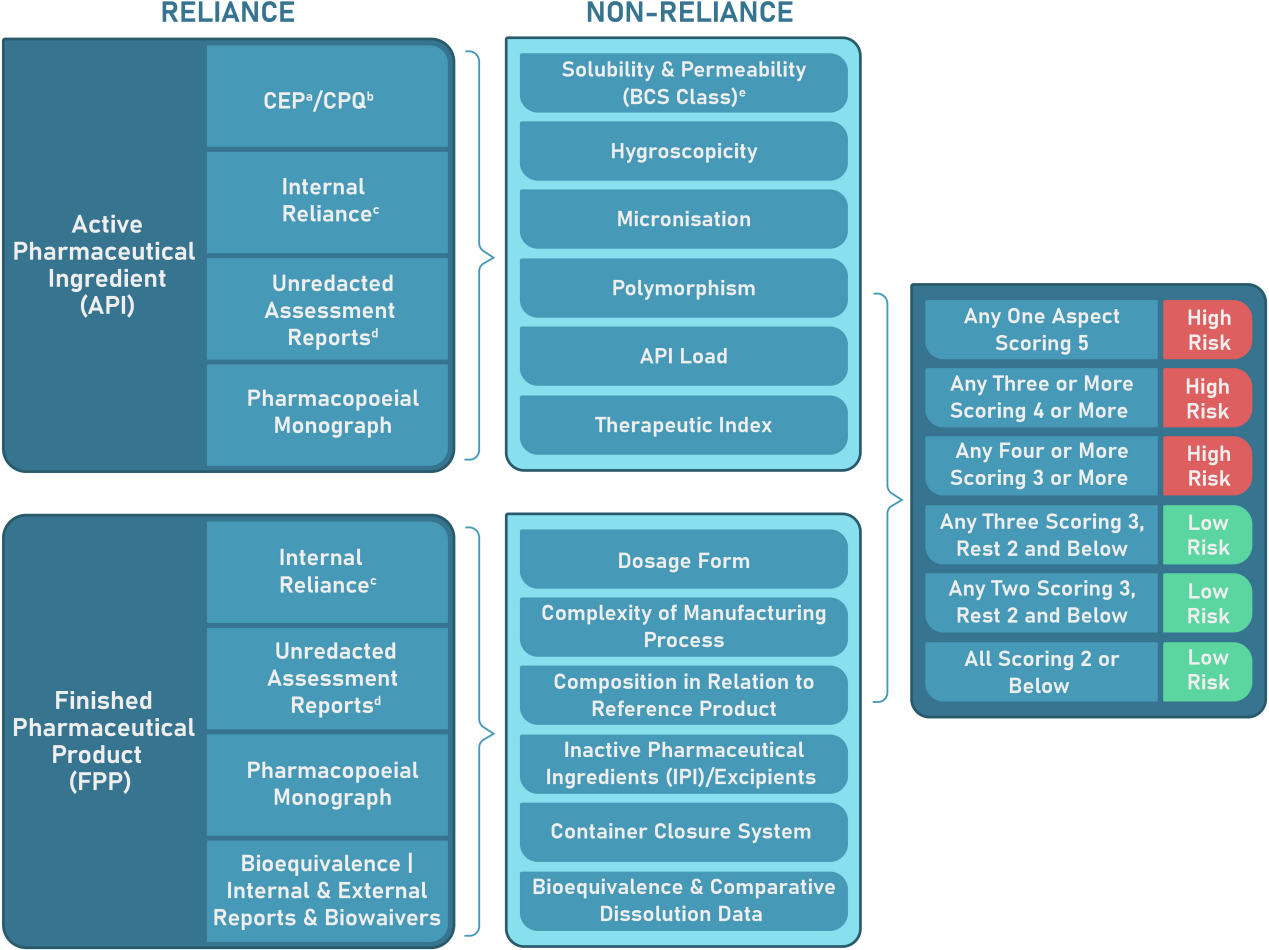
A simplified SAHPRA Backlog Clearance Project (BCP) risk-weighting schematic developed for generic products [adapted from Moeti et al. (11)]. *^a^*Valid Certificates of Suitability (CEPs), issued by the European Directorate for the Quality of Medicines (EDQM); *^b^*Valid Certificates of Prequalification (CPQs), issued by the WHO Prequalification Team: Medicines (WHO PQTm); *^c^*The practice of relying on previous product assessments performed by the NRA itself; *^d^*Unredacted assessment reports from reference agencies that an NRA trusts and aligns with; *^e^*Biopharmaceutics Classification System (BCS) (33), in particular aqueous solubility.

When the previous South African medicines regulator, the Medicines Control Council (MCC), transitioned into the South African Health Products Regulatory Authority (SAHPRA) in 2018, the new authority inherited approximately 16,000 backlogged new marketing authorization and variation applications—a direct result of the MCC’s lengthy review times (1, 2). During the period between 2011 and 2017, it took the MCC a median of 2,092 calendar days to authorize a new product and a median of 1,622 days from 2015 to 2018 (10, 11). To address the accumulated pipeline of waiting applications, SAHPRA, through its Backlog Clearance Project (BCP), piloted the implementation of global best regulatory assessment practices, such as reliance (1, 2) and risk-based assessment (11), the latter for the CMC properties of generic products. The assessment of CMC or quality-related product characteristics had been a key contributor to the delays in the approval of generic medicines by SAHPRA (11). During the BCP, a full review of the CMC and bioequivalence (BE) information (as the latter data were reviewed by the same unit within SAHPRA) (11) took a median of 272 days, with the CMC data assessment for some products taking as long as 495 days (1). To mitigate the lengthy assessment time, SAHPRA initiated an RBA pathway, which focused on the classification of the particular risk a product posed to the end-user, the patient. Prior to the evaluation of a product application, an expert assessor completed a manual risk-weighting of the product, using a scoring template and assigning a numerical value of 1 (low risk) to 5 (high risk) to each CQA (11). Reliance on prior work conducted by reference agencies and institutions with which SAHPRA aligns (12), a stratagem SAHPRA only fully introduced during its BCP (11), as well as internal reliance, where SAHPRA had previously assessed the APIs and/or FPPs, lowered the risk apportioned to a product (11). Figure 1 further details the areas, based on specifically identified CQAs, that were considered during the product pre-evaluation risk-weighting step (11).

The experimental RBA approach proved pivotal to clearing the significant queue of especially new multisource products that awaited authorization, with Moeti et al. reporting that the CMC/BE assessment of the generic applications used in the pilot was performed within 68 calendar days (11).

This kind of upfront manual risk-weighting by reviewers can, however, be time-consuming and is susceptible to reviewer bias, despite the provisos of an RBA framework. For this reason, it is envisioned that leveraging AI will enable faster, more efficient risk-scoring of generic medicines. AI and ML have emerged as powerful drivers of innovation across diverse domains, enabling new advances in data analytics and automation. Within the pharmaceutical sector, their adoption is especially critical, offering a considerable opportunity to automate regulatory workflows and enhance both the accuracy and speed of decision-making, translating into greater accessibility to medicines.

The aim of this research was, therefore, to develop a bespoke RBA AI proof-of-concept (PoC) tool, built on the tenets of the risk-based assessment practices piloted within SAHPRA’s BCP, and to determine this algorithm’s applicability to the proposed use case, as well as its ability to establish an accurate risk-weighting and an extraction of the salient quality aspects of a generic product to be reviewed. A final objective was to examine the relevance and advantages of integrating AI into the medicines regulatory space, while acknowledging the risks that such integration may entail.

The objectives of this study were to:

1. In conjunction with SAHPRA and an identified software vendor, develop an AI tool to automate the risk-weighting of generic products submitted for NRA authorization.
2. Verify the accuracy of the developed tool, that is, the algorithm outputs compared with the manual risk-scoring by expert assessors, through a robust validation process within two African NRAs.
3. Evaluate the feasibility, predictive performance, and potential efficiency gains of the AI-assisted RBA PoC tool for supporting quality dossier triage across different African regulatory settings.

## 2 Materials and methods

### 2.1 Development of a risk-based assessment artificial intelligence tool

A key objective of this study was the development of a tailored algorithm designed to automate manual, evaluator-directed risk determinations for generic products within SAHPRA. To initiate this effort, a request for proposals (RFP) was issued to software development firms with demonstrated AI expertise within the African medicine regulation sphere, supported by external funding for SAHPRA in this endeavor.

The RFP outlined the conceptual framework of the RBA AI PoC tool, its intended use case, and the plan for testing the tool using a representative sample of SAHPRA product dossiers. To ensure technical and operational feasibility, the following minimum requirements for prospective developers were specified:

1. Demonstrated completion of at least one prior AI project.
2. Availability of qualified AI experts who could be mobilized promptly and remain engaged throughout the project’s duration and validation of the developed tool in two NRAs.
3. A history of prior grants or contracts with the funding partner.
4. Capacity to deliver within the specified budget envelope and fixed project timelines.
5. Provision of open access of the algorithm to any NRA wishing to utilize its capabilities.

In addition to these requirements, desirable attributes for potential vendors included a location on the African continent (preferably South Africa), prior experience collaborating with an NRA, and a minimum 5-year track record in implementing digital projects in Africa. Applicants had to submit both a technical and financial proposal to be considered.

Upon selection, the successful firm had to develop an algorithm based on the SAHPRA quality risk matrix, which identifies and rank-orders the various risk-associated attributes and captures all relevant permutations for generic products. In this the developer was assisted by the two SAHPRA expert CMC assessors who had led the RBA pilot during the BCP. Furthermore, it was anticipated that the reports on more than 200 product applications that had been manually risk-weighted and assessed during SAHPRA’s pilot studies would be utilized in the creation of the tool, with the final tool testing and validation with “human assessors-in-the-loop” to assure the acceptable accuracy of the AI tool.

### 2.2 Validation of the risk-based assessment artificial intelligence proof-of-concept tool

Once developed, determining the accuracy of the RBA AI tool, compared to the manual risk-weighting of NRA expert quality assessors, was imperative. Therefore, validation of the tool was conducted in strict accordance with GAMP (Good Automated Manufacturing Practice) guidelines, specifically following the International Society for Pharmaceutical Engineering (ISPE) GAMP5 Guide: A Risk-Based Approach to Compliant Good Practices (GxP) Computerized Systems, Second Edition (13). GAMP5 provides a structured, risk-based methodology that ensures computerized systems in regulated industries like pharmaceuticals and medical devices are fit for their intended use while maintaining compliance with regulatory requirements and protecting patient safety, product quality, and data integrity. GAMP5 also “facilitates the effective and efficient use of valuable resources by the application of appropriate and proportionate practices, encouraging innovative approaches to managing risk to patient safety, product quality, and data integrity, while supporting benefit to public health” (13).

**FIGURE 2:**
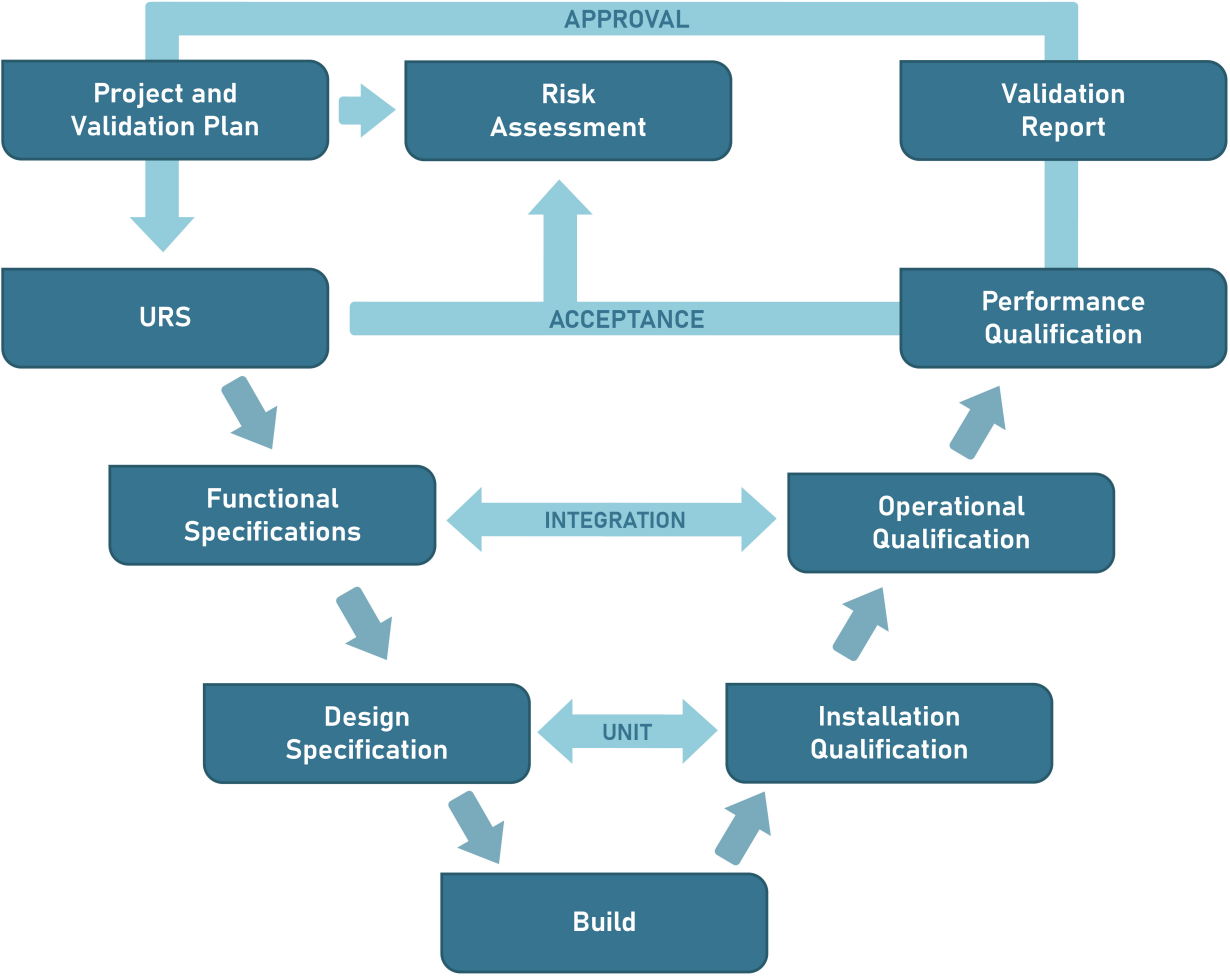
Good Automated Manufacturing Practice validation flow-diagram, adapted from ISPE: GAMP^®^5 (13). The left side of the “V” represents the User Requirements Specifications (URS), functional specifications and design, with the bottom of the “V” denoting the build or development phase. The right side of the “V” depicts the testing and qualification (IQ, OQ, PQ) activities, i.e., the validation of the tool, and the cross-links show how each requirement/specification must be verified during testing.

This development phase of the algorithm was divided into three validation sub-phases (Figure 2), namely:

1. Installation Qualification (IQ): Verifying the software installation on target hardware and operating system and documenting the installation procedures and configuration settings,
2. Operational Qualification (OQ): Executing the test scripts and scenarios to validate software functionality, and
3. Performance Qualification (PQ)/User Acceptance Testing (UAT): Testing the software with real-world data and scenarios (13).

Once the validation had been successfully completed, the aim was to determine the functionality and effectiveness of the algorithm within two African NRAs to ensure adaptability and interoperability of the tool in different production environments (external validation).

### 2.3 Ethics Approval

The study was approved by Health, Science, Engineering and Technology ECDA, University of Hertfordshire, United Kingdom (Reference Protocol number: 1042 2025 May HSET). The authors confirm that no human or patient data were used and that the AI use complied with data privacy and governance frameworks. The study involved the use of regulatory product dossiers containing CMC information only, with no human or patient-level data included. Data transfers were conducted under formal Non-Disclosure Agreements between the participating NRAs and the developer. The dossiers were stored in a secure, access-controlled environment, with access restricted to authorized personnel, including designated NRA expert assessors and the development team. Data were processed within controlled infrastructure to ensure data residency and confidentiality. As the system was designed to evaluate product quality attributes, relevant technical and manufacturing information was retained; however, no personally identifiable or patient-sensitive data were used at any stage.

## 3 Results

### 3.1 Development of a risk-based assessment artificial intelligence tool

**FIGURE 3:**
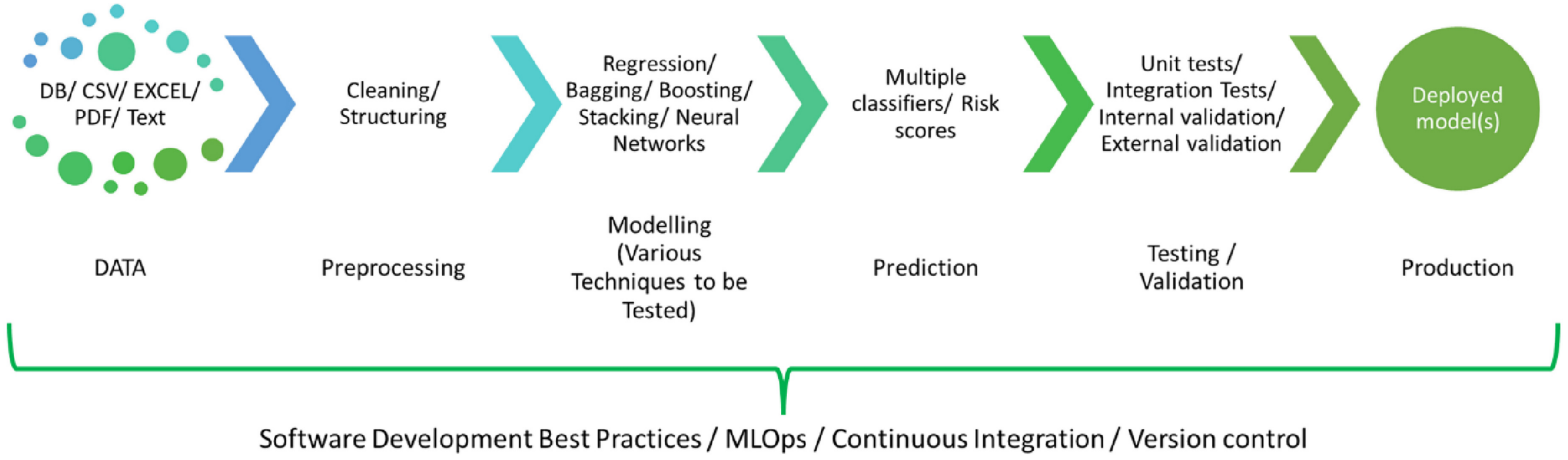
Scigenix project approach for the development of SAHPRA’s RBA AI PoC Tool.

In response to the published RFP, Scigenix (Pty) Ltd. (14), a South African-based software developer, was identified after successfully demonstrating their experience in the development and deployment of digital and AI/ML projects in the African public health space. The developer envisioned that, through development, testing, validation, user training, and ongoing support, the project approach detailed in Figure 3 aimed to deliver a seamless and efficient AI risk weighting tool that caters to SAHPRA’s unique requirements and goals.

### 3.2 Construction of the AI tool

The construction of the AI tool, based on the SAHPRA BCP-generated RBA matrix, commenced in November 2023, and followed the process steps of:

1. Project initiation and scoping,
2. Requirements gathering,
3. AI tool planning, which included the establishment of data-sharing, data protection and privacy agreements with access to historical SAHPRA data and regulatory knowledge, crucially required for training and validating the algorithm.
4. AI tool development,
5. Testing and validation of the tool, which comprised of the testing during the pilot proof-of-concept (POC) program, as well as testing during the formal development phase. This involved two stages: (1) internal validation of performance during the development of the AI tool, (2) external validation by two experienced SAHPRA RBA assessors, and (3) a final external validation in a second NRA locale,
6. User training,
7. Ongoing monitoring and maintenance, and
8. Project closure.

Following preliminary evaluations, the use of neural networks, specifically Retrieval-Augmented Generation (RAG), was selected as the approach to achieve the required results. RAG is capable of rapidly querying the large amount of unstructured text data captured in a complete dossier using embeddings and vector databases. The RBA AI PoC tool, which was nicknamed LEXI (Linguistic Expert), was developed over the course of 18 months, using the Large Language Model (LLM), Llama 3.1, with in-depth regulatory support provided by SAHPRA. The evolution of the LEXI technology stack—a collection of technologies, tools, frameworks, and programming languages used in conjunction to build and run a software application or digital system (15)—is detailed in Supplementary Table 1 in terms of its main layers.

### 3.3 System architecture and technology stack

LEXI was implemented using Meta’s Llama 3.1 (8B parameter model), deployed locally via the Ollama framework to maintain full control within the NRA’s secure environment. The system operates on Python 3.9, containerized using Docker to ensure workflow consistency, dependency management, and secure isolation of all processing components.

This combination of technologies provides a scalable and secure environment for AI-assisted regulatory evaluation. To enhance transparency and reproducibility, a model card–style summary of the LEXI system configuration, including data sources, preprocessing workflows, model components, and validation datasets, is provided in Supplementary Table 2.

### 3.4 Retrieval-augmented generation (RAG) framework

LEXI’s RAG framework connected the LLM to a structured knowledge base of regulatory documentation, that is from “narratives to structure.” Application dossiers were vectorized into high-dimensional embeddings, enabling semantic retrieval of relevant content even when phrasing varied from standard terminology. The retrieval corpus comprised keyword-filtered text from applicant-submitted dossier PDFs and structured reference data from internal reliance worksheets. No assessor-generated outputs, final risk classifications, or validation labels were included in the indexed data. A strict separation was maintained between the retrieval corpus and the expert-derived reference classifications used for validation to prevent information leakage. Upon receiving a query, the system retrieved pertinent context from its indexed knowledge base and used this to guide the LLM’s reasoning, thereby grounding all AI-generated outputs in the provided regulatory texts.

This architecture ensured that the AI’s outputs reflected domain-specific knowledge rather than generalized LLM patterns, addressing one of the main limitations of traditional AI approaches in highly specialized regulatory contexts.

### 3.5 Document intelligence and content processing

The document processing pipeline was designed to accommodate the heterogeneity of pharmaceutical submissions, including scanned PDFs, multi-column layouts, and embedded tables.

Using OCRMyPDF configured for 150 dots per inch (DPI) and executed with parallel processing on the central processing unit (CPU), LEXI achieved high-fidelity text extraction while maintaining computational efficiency.

An intelligent classification layer automatically recognized document types (e.g., Common Technical Document (CTD) Modules 3.2.S and 3.2.P) and routed them through appropriate processing workflows. A two-stage filtering mechanism identified relevant sections based on regulatory codes and then searched for domain-specific terminology within those sections. Text extraction was performed using a keyword-anchored character-window approach (approximately 100–300 characters), with adaptive context expansion where needed. Retrieval was implemented using cosine similarity with a top-k of 2 within the LlamaIndex framework. Tables were converted to text during parsing, and OCR was applied for scanned documents.

### 3.6 Integration of external regulatory data

LEXI also incorporated structured data from authoritative external sources to enhance contextual accuracy, including the:

1. European Directorate for the Quality of Medicines & HealthCare (EDQM) Certificate of Suitability (CEP) database (8),
2. World Health Organization (WHO) Prequalification listings (9), and
3. DrugBank (16).

These integrations were achieved through automated web scraping using browser automation with intelligent retry logic, enabling real-time access to reference data without dependence on external application programming interfaces (APIs) or data-sharing agreements.

### 3.7 Modular criterion processing and traceability

Each risk criterion within LEXI (detailed in Table 1) functioned as an independent module following a standardized workflow:

**TABLE 1:** Description of individual critical quality attributes (CQAs) assessed.

Abbreviation	Description
RA1	API reliance (CEP/CPQ submission, internal and external reports)
RA2*	API specifications (pharmacopoeial or in-house)
RF1	FPP reliance (internal or external reference agency reports available)
RF2*	FPP specifications (pharmacopoeial or in-house)
RB1	FPP bio-equivalence (BE) (internal SAHPRA reliance on previous biostudy reports assessed, or assessments by reference agencies)
A1	API solubility and permeability (BCS class)
A2	API hygroscopicity (solid oral dosage forms)
A3	API particle size (solid oral dosage forms)
A4	API polymorphism (solid oral dosage forms)
A5	API load (concentration) (solid oral dosage forms and semi-solids)
A6	API therapeutic index (all dosage forms)
F1	Type of dosage form as per dosage form classification (32)
F2	Complexity of the manufacturing process
F3	Composition in relation to the comparator product
F4	Excipients
F5	Container closure system
B1	Bioequivalence and comparative dissolution with the comparator products/biowaivers

*This criterion was only assessed during the internal validation phase and was not included in the final validation at SAHPRA or BoMRA.

1. Retrieve relevant data from local databases.
2. Filter and extract pertinent information from submission documents.
3. Apply criterion-specific reasoning through engineered prompts.
4. Generate traceable and reproducible outputs.

The modular design allowed continuous refinement of individual criteria, as well as easy expansion of the tool, without affecting system stability.

Comprehensive logging and audit trails recorded each processing decision, ensuring that all outputs were fully traceable to source documents and reasoning steps. This transparency was crucial for maintaining regulatory accountability and supporting validation audits.

### 3.8 Security and deployment environment

All components of LEXI were encapsulated within a Docker container, ensuring controlled deployment across diverse environments. Local hosting of the language model and data processing pipeline prevented the transfer of any confidential regulatory data to external servers or cloud services (this being an internal SAHPRA decision; however, the tool may also be deployed to the cloud). This security-centric design met the NRA’s data protection requirements and allowed independent verification of software integrity.

### 3.9 Training, testing, and validation

LEXI’s development and testing involved the complete set of 210 regulatory dossiers previously assessed during the RBA pilots under SAHPRA’s BCP. A subset of 157 dossiers (75%) was used for system development and calibration, including prompt design, rule definition, and iterative refinement of the retrieval and scoring workflows, while the remaining 53 dossiers (25%) were reserved for internal validation. The dataset was split using a random allocation approach. The AI-generated risk assessments were cross-verified by the SAHPRA expert assessors, confirming consistency between the automated and manual determinations. This internal validation phase demonstrated the system’s reliability as a decision-support tool capable of augmenting, but not replacing, expert judgment.

### 3.10 Good automated manufacturing practice validation framework implementation

LEXI was classified as a Category 5 (Custom or Bespoke software) under the GAMP5 framework (13). This categorization was determined through a comprehensive risk assessment using Scigenix’s internal Quality Management System that evaluated the system’s complexity, novelty, and potential impact on product quality and patient safety. As an AI-driven tool for regulatory decision support, LEXI required robust validation to ensure consistent and reliable risk assessments of generic pharmaceutical products. The GAMP5 validation lifecycle was implemented through three sequential qualification phases, as was detailed under Materials and methods:

*Installation Qualification (IQ)*—The IQ phase verified that LEXI was correctly installed and configured in the target environment, including system architecture verification (Docker containerization, Python 3.9.x environment, Ollama framework, Llama 3.1 model, and essential Python packages), infrastructure requirements validation, security configuration confirmation, and documentation completeness verification.

*Operational Qualification (OQ)*—The OQ phase validated LEXI’s functional capabilities against design specifications through comprehensive testing of document processing capabilities, external data integration, RAG functionality, multi-API and multi-manufacturer processing, dosage form recognition, scoring logic implementation, and data integrity and traceability features.

*Performance Qualification (PQ)/User Acceptance Testing (UAT)*—The PQ phase tested LEXI’s performance against real-world operational requirements using 30 SAHPRA dossiers submitted outside of the BCP. A sample size of 30 dossiers was selected for external validation, balancing the need for independent performance assessment with the resource-intensive nature of expert-led manual review, and is consistent with PoC validation studies in similar contexts. Each dossier was independently assessed by two experienced SAHPRA evaluators using the established manual risk-weighting methodology, with assessors blinded to the LEXI-generated outputs during their initial evaluations. Following completion of the independent assessments, discrepancies were identified and reviewed. Where differences arose, a consensus-based adjudication process was undertaken, whereby the assessors jointly reviewed the dossier evidence and applied the predefined RBA criteria to reach a final agreed classification. The adjudicated consensus outcomes were used as the reference standard for comparison with LEXI predictions. Vendor personnel had no role in setting acceptance thresholds or gold standard labels for validation datasets.

### 3.11 Quality assurance framework

The validation process maintained rigorous documentation standards including comprehensive traceability matrices mapping all requirements to test cases, formal deviation management procedures, change control processes for code updates, and systematic evidence collection through screenshots, log files, and Excel outputs.

### 3.12 Validation results

#### 3.12.1 South African Health Products Regulatory Authority

The interim validation results in terms of the individual CQAs (Table 1) highlighted variances in the risk stratification tool, as well as irregularities in evaluators’ feedback. In some instances, discrepancies were observed between initial assessor classifications prior to consensus adjudication, reflecting variability in interpretation of certain criteria (notably RA2 and RF2), including questions not deemed relevant and subsequently skipped by the assessors. Moreover, LLMs, which enable systems to understand, interpret, and generate human language, are not well-suited for directly handling mathematical equations as these are symbolic, not linguistic. This affected the calculation of A5 (API load), with a subsequent incorrect risk-weighting of the CQA concerning the therapeutic index of the active pharmaceutical ingredients (A6). Criteria F1 and F2, which pertain to the scoring of the dosage form and the complexity of the FPP manufacturing process, respectively, also required clearer delineation, and testing with a wider set of product dosage forms. Fixed-dose combinations (FDCs) were, for example, not part of the 210 dossiers used for the testing and validation of LEXI. Despite these initial deficiencies during internal validation, the accuracy of LEXI for the individual risk criteria, and for the dossier as a whole, was still 81 and 80%, respectively, with an initial estimated average time saving of 91% in determining the risk areas of a generic product.

**FIGURE 4:**
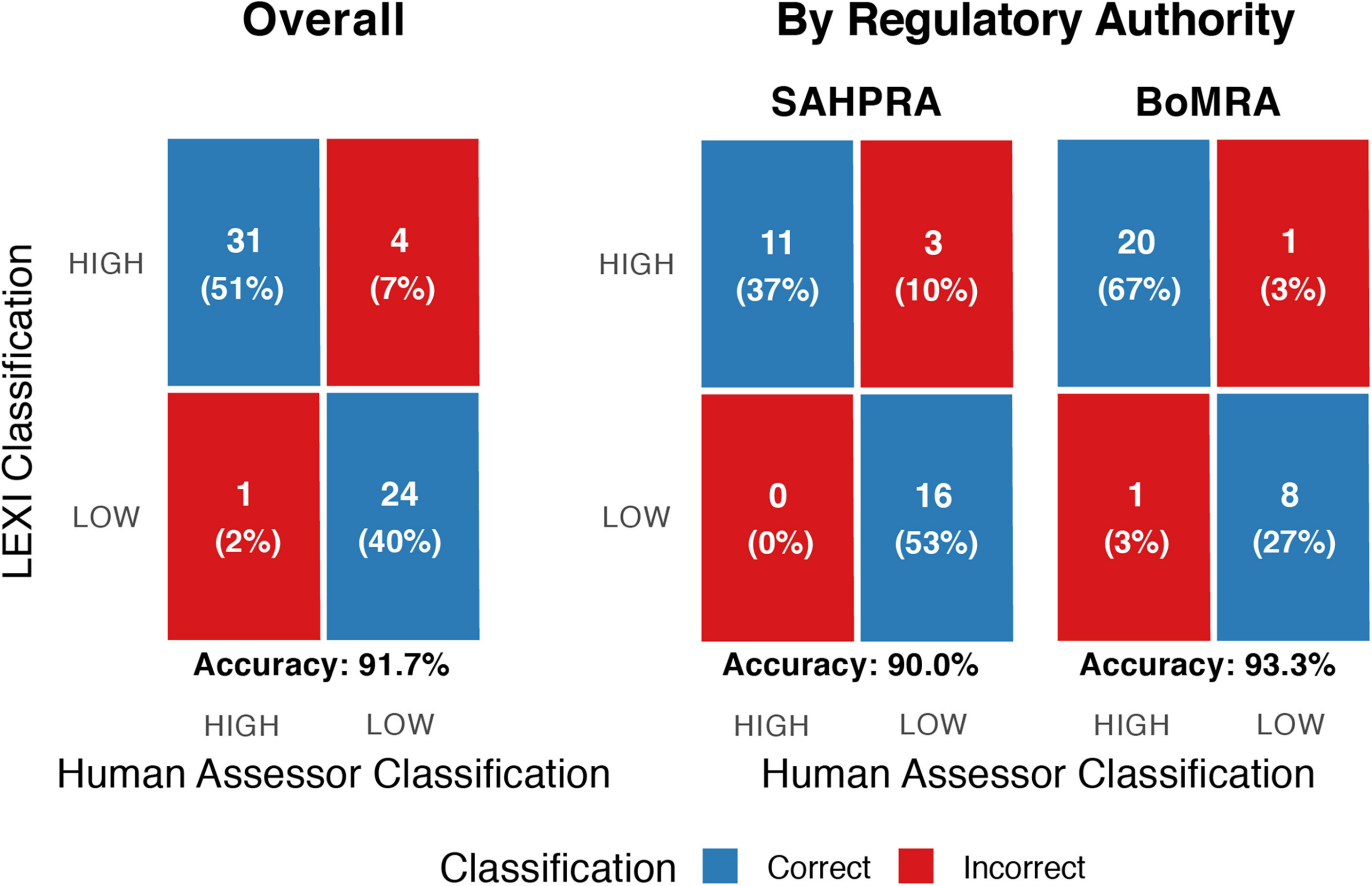
LEXI prediction accuracy on external validation—overall and stratified by regulatory authority. The rows represent the LEXI predictions and columns represent the reference (human assessor-adjudicated) classifications. The confusion matrix is based on 60 dossiers, comprising 32 high-risk and 28 low-risk cases. Overall performance includes 31 true positives, 24 true negatives, 4 false positives, and 1 false negative.

However, due to these discrepancies, 20 PQ tests were added to the Error Log (details included in Supplementary Table 3), which required the tool to undergo further updates and a revalidation of the iterated version against standardized evaluator feedback – this external validation was done utilizing 30 SAHPRA dossiers not evaluated during its BCP. The outcome of the validation established a stringent 90% accuracy threshold as the acceptance criterion (Figure 4).

#### 3.12.2 Botswana Medicines Regulatory Authority

As a final step, LEXI underwent external validation at a second NRA, the Botswana Medicines Regulatory Authority (BoMRA); this NRA was selected as (1) it is experiencing a backlog of generic products, with a subsequent unavailability of medicines in the country and (2), of more importance, its Board has endorsed a strategy with a strong focus on AI-driven optimization of its workflows. During the validation of the tool with 30 BoMRA dossiers (which included a number of fixed-dose combination products), it was observed that applicants, when submitting to this authority, had not adhered to the approved ICH naming conventions required by both the ICH eCTD Specification v3.2.2 (Appendix 3) (17) and the ICH eCTD v4.0 Implementation Guide (section 5.2) (18).

In contrast, SAHPRA mandates compliance with ICH naming conventions (19), as applicants are required to submit dossiers in the electronic CTD (eCTD) format via the Lorenz DocuBridge submission platform. As the eCTD format has yet to be introduced at BoMRA, the agency has not issued specific guidance instructing applicants to align with the ICH requirements for file and folder naming. File and folder names in the BoMRA dossiers frequently contained spaces, periods, capital letters, and spelling inconsistencies—none of which are permitted by the official ICH eCTD specification documents. In one instance, among the thirty dossiers received, a single file name (“2.3 Quality Overall Summary”) appeared in 11 ICH-divergent variations.

However, due to the modular design of the system, Scigenix was able to modify the tool to accommodate the file and folder naming patterns used by BoMRA’s applicants. The developer concurrently incorporated both the correct eCTD naming conventions and the BoMRA-specific variations, ensuring continued eCTD compliance. Moreover, due to the lack of eCTD formatting, CTD Module 2 was not being properly accounted for, and a minor additional change was required to accommodate BoMRA’s formatting. These adjustments resulted in an additional deviation being documented in the Verification and Testing and Validation Summary reports. A formal ablation analysis to quantify the impact of dossier structure and naming variability on system performance was not conducted in this study. However, observations from the BoMRA validation suggest that deviations from standardized submission formats can influence document interpretation workflows and future work should include controlled evaluations to quantify the impact of such variability on AI-assisted regulatory tools.

### 3.13 External validation predictive performance of the AI-based risk classification tool

Across the 60 externally validated dossiers (32 high-risk, 53.3%; 28 low-risk, 46.7%), LEXI achieved an overall predictive accuracy of 91.7% (55/60; 95% CI: 81.6–97.2%) relative to human assessor classifications. Overall sensitivity was 96.9% (31/32; 95% CI: 83.8–99.9%), and overall specificity was 85.7% (24/28; 95% CI: 67.3–96.0%) (As several subgroup estimates were based on small sample sizes and proportions close to 0 or 1, exact binomial 95% confidence intervals were calculated using the Clopper–Pearson method) (20). The model yielded a median time reduction of 91%, although performance varied by criterion and dossier structure. The confusion matrix (Figure 4) revealed that the model correctly classified 31 of 32 high-risk dossiers (∼97% sensitivity) and 24 of 28 low-risk dossiers (∼86% specificity). Misclassifications included 4 dossiers incorrectly predicted as high when assessed as low by human evaluators (7% false positive rate) and 1 dossier incorrectly predicted as low when assessed as high (2% false negative rate). When stratified by regulatory authority, the model demonstrated comparable performance across both jurisdictions. For the SAHPRA dossiers (*n* = 30), the accuracy was 90.0% (27/30; 95% CI: 73.5–97.9%), with perfect sensitivity of 100.0% (11/11; 95% CI: 71.5–100.0%) and specificity of 84.2% (16/19; 95% CI: 60.4–96.6%). No false negatives were observed in the SAHPRA subset, though 3 low-risk dossiers were incorrectly classified as high-risk (10% false positive rate).

For the BoMRA dossiers (*n* = 30), the accuracy was 93.3% (28/30; 95% CI: 77.9–99.2%). The model correctly identified 20 of the 21 high-risk dossiers (95.2% sensitivity; 20/21; 95% CI: 76.2–99.9%) and 8 of the 9 low-risk dossiers (88.9% specificity; 8/9; 95% CI: 51.8–99.7%). One high-risk dossier was misclassified as low (3% false negative rate), and one low-risk dossier was misclassified as high (3% false positive rate).

### 3.14 CQAs predictive performance

**FIGURE 5:**
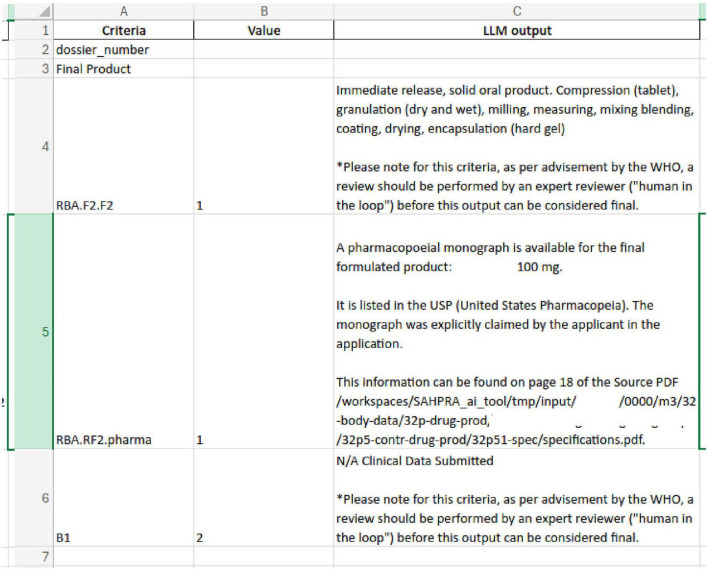
An example abstract of de-identified LEXI output demonstrating criterion-level risk classification, supporting evidence extraction, and traceability to source dossier content. The output includes structured scoring for individual criteria, explanatory text generated by the model, and references to source documents, illustrating how assessors can review and verify system outputs within the regulatory workflow.

**FIGURE 6:**
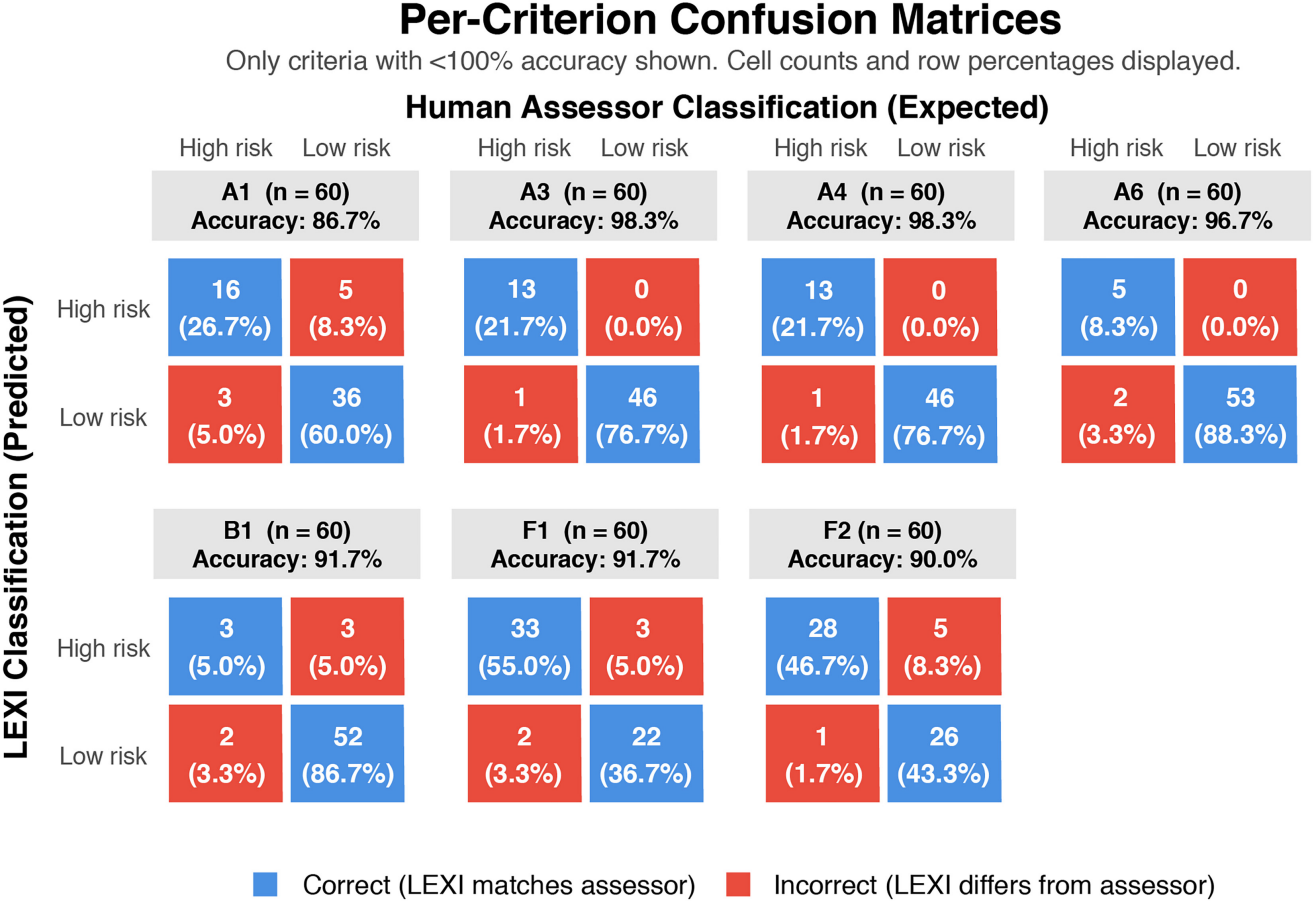
Criterion-level confusion matrices for LEXI predictive performance across all dossiers (*n* = 60). Only criteria with <100% accuracy are shown. Cell counts and percentages are displayed, with rows representing LEXI predictions and columns representing reference (human assessor-adjudicated) classifications. Blue cells indicate correct classifications and red cells indicate misclassifications. Accuracy for each criterion is shown above each matrix. Criteria definitions are provided in Table 1.

To examine the AI tool’s performance at a more granular level, Scigenix analyzed the prediction accuracy for the individual risk classification criteria; an illustrative example of a de-identified LEXI output, including criterion-level scoring, supporting evidence, and traceability to source documents, is presented in Figure 5. Across all 900 criterion-level classifications (15 unique criteria evaluated across 60 dossiers), eight criteria achieved perfect accuracy (100%), while seven criteria showed some degree of misclassification. Figure 6 presents confusion matrices for these seven criteria where the accuracy was less than 100%.

Among the imperfectly classified criteria, accuracy ranged from 86.7% to 98.3%. The API-related criteria (A1, A3, A4, A6) demonstrated high accuracy, with A3 and A4 both achieving 98.3%. Criterion A1 showed the lowest accuracy at 86.7%, with 5 false positives (low-risk criteria incorrectly classified as high, 8.3%) and 3 false negatives (high-risk criteria incorrectly classified as low, 5.0%). Criterion A6 achieved 96.7% accuracy with no false positives and 2 false negatives (3.3%).

The bioequivalence criterion (B1) achieved 91.7% accuracy, with 3 false positives (5.0%) and 2 false negatives (3.3%). Among the final pharmaceutical product criteria (F1, F2), F1 demonstrated 91.7% accuracy with 3 false positives (5.0%) and 2 false negatives (3.3%), while F2 achieved 90.0% accuracy with 5 false positives (8.3%) and 1 false negative (1.7%). This pattern suggests the model was more conservative for FPP criteria, tending to over-predict high-risk classifications rather than under-predict them.

### 3.15 CQA-level performance across national regulatory authorities (NRAs)

**FIGURE 7:**
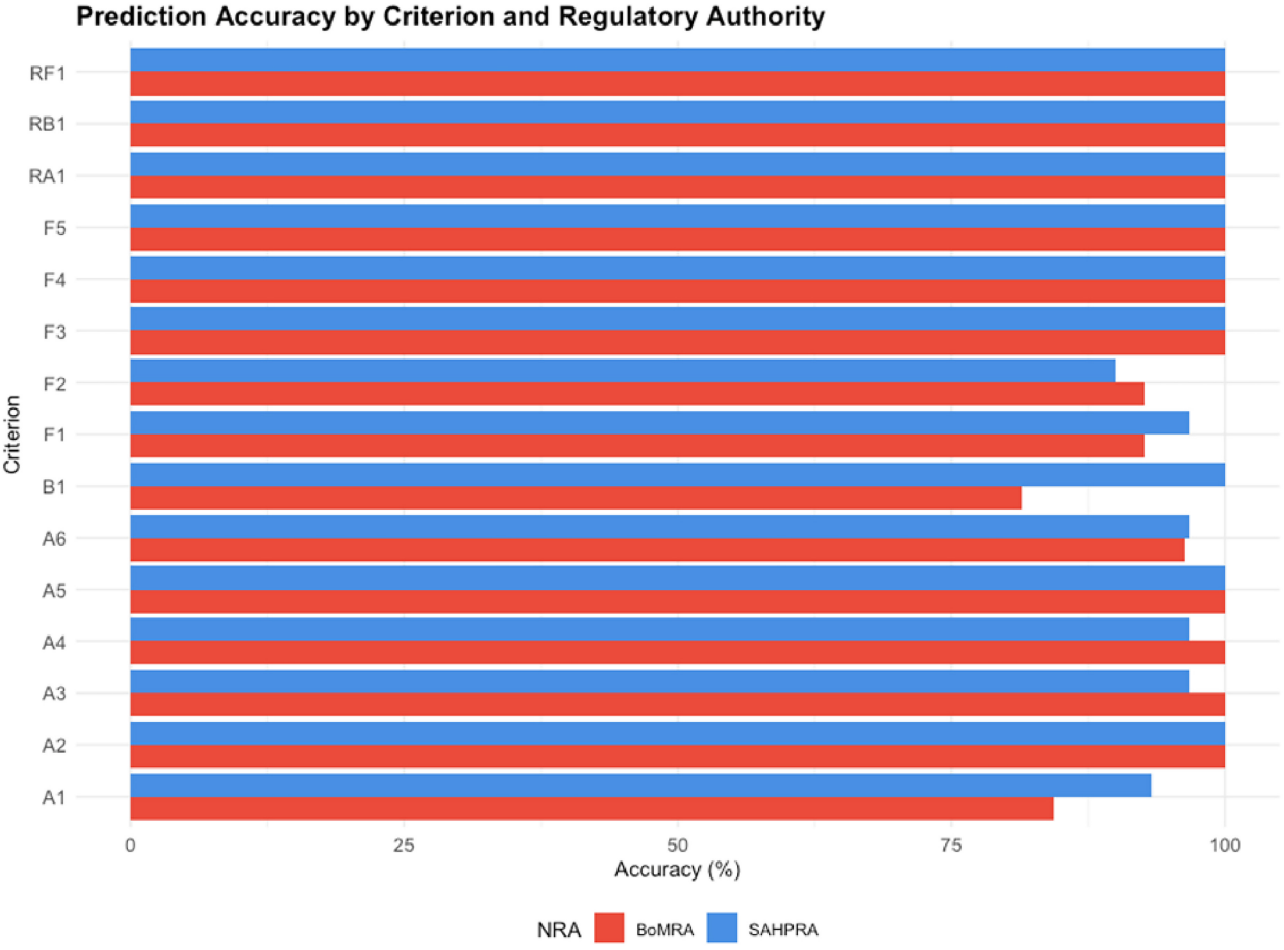
Criterion-level prediction accuracy of LEXI stratified by regulatory authority (SAHPRA and BoMRA). Accuracy represents the proportion of dossiers correctly classified for each criterion (*n* = 30 per NRA). Performance is shown for all evaluated criteria, highlighting variability across criteria and between regulatory contexts.

To assess whether prediction accuracy varied by regulatory context, the tool developer compared criterion-level performance between the SAHPRA and the BoMRA submissions (Figure 7). The model demonstrated comparable performance across both regulatory authorities, with most criteria showing similar accuracy levels between the two jurisdictions. For the SAHPRA dossiers, criterion-level accuracy was consistently high, with 13 of 15 criteria achieving ≥ 95% accuracy. The model performed particularly well on reliance-based criteria (RA1, RB1, RF1) and most FPP criteria (F3, F4, F5), all achieving 100% accuracy. Lower accuracy was observed for criterion A1 (93%) and F2 (90%). For the BoMRA dossiers, accuracy was similarly high across most criteria, though with a slightly different pattern of variation. Criteria A2, A3, A4, and A5 showed high accuracy (≥95%). However, A1, B1, and F1 showed notably lower accuracy with 80, 83, and 87%, respectively, representing the poorest criterion-level performances across both regulatory authorities.

The most pronounced difference between the regulatory authorities was observed for criterion B1 (bioequivalence), where the SAHPRA accuracy was approximately 20% higher than BoMRA accuracy. Conversely, criteria A3 and A4 showed the opposite pattern, with BoMRA demonstrating a slightly better performance than SAHPRA. For the majority of criteria, however, inter-regulatory agency differences were minimal (≤5%), suggesting the model’s predictions were largely robust to regulatory context.

## 4 Discussion

The advent of AI tools has introduced opportunities for enhancing regulatory review systems within medicines regulators, with work being done globally to harness the capabilities of AI. There is an expectation that custom-designed AI tools will play a facilitating role in the overall strengthening of medicine regulatory systems going forward, resulting in vital access to medicines to disadvantaged populations, especially in African LMIC settings. Algorithms can, with a high level of accuracy, assume administrative tasks such as technical screening or triaging of product dossiers to enable faster, more competent decision-making by African regulators.

### 4.1 Principal findings and regulatory significance

The Scigenix-developed RBA PoC tool, termed an “Assisted Structured Assessment Tool” by SAHPRA, has shown that an AI system can automatically extract, interpret, and summarize key content while providing intelligent risk stratification to improve prioritization. LEXI combines natural language processing, document intelligence, and regulatory data integration to provide structured, traceable risk analyses to process almost 92% of dossiers efficiently. With this efficiency also comes a timesaving of approximately 91% (1.5-h, utilizing the algorithm, versus the estimated 16-h (2 day) manual process), depending on the complexity of the product. By identifying the high-risk areas of a generic product and guiding NRA assessors to the respective CQAs through a summary produced by the tool, the review timelines are drastically reduced; an expedient in clearing generic application backlogs without compromising on the quality, safety and efficacy of the medicines (11). The development of LEXI demonstrates how advanced LLMs can be adapted for use within tightly controlled regulatory environments. LEXI serves as a decision-support tool that complements human expertise, streamlining the regulatory process while ensuring that expert assessors retain oversight and final decision-making responsibilities remain with the NRA, as per its mandate.

### 4.2 LEXI: future development, reproducibility considerations and governance

A key limitation identified during validation relates to the handling of mathematically derived criteria, such as API load (A5) and therapeutic index (A6), where LLMs are not inherently optimized for deterministic numerical computation. Future iterations of LEXI should, therefore, incorporate dedicated deterministic calculation modules or symbolic computation layers to process quantitative inputs (e.g., dose, strength, and threshold values) outside of the LLM, thereby improving accuracy and reproducibility of these criteria.

The absence of FDCs in the training dataset, furthermore, represents a restriction in terms of the PoC development; however, the system demonstrated adaptability when encountering such dosage forms during external validation. This suggests that the underlying approach is robust but requires further validation across a broader range of product types, dosage forms, and regulatory contexts to confirm generalizability.

In addition, misclassification, particularly false negatives, carries significant potential impact in a regulatory context, as it may lead to insufficient scrutiny of CQAs. As such, subsequent system iterations should incorporate safeguards, such as sensitivity-driven evaluation thresholds and structured escalation mechanisms, to ensure that potentially high-risk dossiers are not under-reviewed.

Further technical refinement should also address the integration of external regulatory data. Future iterations of LEXI should incorporate controlled ingestion of curated and versioned external reference datasets, supported by appropriate snapshotting and audit mechanisms, to ensure data integrity and reproducibility. Consideration should moreover be given to the robustness of external data integrations, including potential failure modes arising from changes in source system structure, availability, or access conditions. Mechanisms such as data inventories, controlled ingestion pipelines, and audit trails will be essential to ensure traceability and reliability. In addition, policies governing system updates and periodic recalibration in response to evolving data distributions will be important to maintain performance and regulatory compliance over time. These considerations are particularly relevant for deployment within regulated public-sector environments, where compliance, data sovereignty, and contractual obligations must be maintained.

Although full disclosure of LEXI’s source code was not feasible because of proprietary components, the system architecture and processing pipeline are described in sufficient detail to support conceptual reproducibility. LEXI produced consistent outputs under defined conditions; however, formal reproducibility testing across repeated executions was not conducted and should be addressed in future evaluations. Future implementations should also consider mechanisms such as source code escrow or controlled-access repositories to enable independent audit and strengthen transparency.

In terms of governance, even though the GAMP5 validation of the LEXI PoC provided strong system-level assurance, contemporary AI governance frameworks, such as ISO/IEC 42001 (21) and the NIST AI Risk Management Framework (22), extend beyond project-level validation and anticipate organization-wide AI management systems to manage risks, ensure accountability and transparency, and maintain appropriate oversight throughout the AI system lifecycle. In the context of emerging AI governance frameworks, key elements of responsible AI implementation can be partially mapped to the current LEXI architecture. Data protection is ensured through local deployment and controlled access to regulatory data, supporting data sovereignty requirements. Human-in-the-loop oversight is maintained, with LEXI outputs serving as decision-support inputs while final regulatory determinations remain with expert assessors. However, additional governance controls, including model drift monitoring, incident response procedures, formal change control processes, and systematic bias evaluation, have not yet been implemented, as the system remains at a proof-of-concept stage. These elements will be essential for operational deployment and alignment with comprehensive AI governance frameworks; future work should include formal mapping of system functionality to established AI governance standards.

### 4.3 Positioning within the evolving global regulatory landscape

In high-income countries, regulatory agencies such as the European Medicines Agency (EMA) (23–25), the UK’s Medicines and Healthcare products Regulatory Agency (MHRA) (26), and the US FDA (27) are similarly exploring and adopting AI tools to improve their regulatory efficiency and outcomes. The EMA published its “Guiding principles on the use of large language models in regulatory science and for medicines regulatory activities” in August 2024 (23), with a reflection paper on the use of AI during a medicine’s lifecycle the following month (24). The EMA followed this with a horizon-scanning exercise to further determine the active and emerging AI/ML use cases in the medicines regulatory sphere (25). Likewise, the MHRA is increasingly turning its attention to AI tools to enhance its role as a “public service organization delivering time-critical decisions”(26). In a further step, the US FDA, after a successful pilot with its assessors, launched “Elsa”—a Generative AI tool to “optimize [its] performance for the American people” in June 2025 (27). Elsa is an LLM-based algorithm adept at summarizing large amounts of data and was built within a high-security internal government cloud, thereby ensuring data security (27). According to the US FDA, the introduction of AI is “a dynamic force enhancing and optimizing the performance and potential of every employee” (27).

Importantly, these regulators are mindful of the ethical, legal, and social considerations tied to AI deployment, with issues such as transparency, explainability, algorithmic bias, data privacy, and public trust actively being addressed through stakeholder consultations and regulatory guidance development. The EMA is proactive in providing high-level guidance to its Member States regarding the introduction of various general-purpose LLMs and how to navigate the pitfalls associated with the models, such as hallucinations, lack of tool training when encountering new data (for example, new active substances), as well as the difficulty in validating these tools to facilitate the safe, responsible and effective use of LLMs (23).

When the lens turns to Africa and its medicines regulators in terms of the future adoption of AI tools, there is an additional set of obstacles to be countered; reliable access to electricity and high-speed internet is limited, thus precluding AI/ML assisted process optimization (28). Ultimately, according to Etori et al., optimal implementation of AI in healthcare “depends on the availability and dependability of the infrastructure,” with the same authors highlighting other “man-made issues” that may hinder the progression of AI implementation in Africa. These include the lack of political will, as well as financial and technical resources to enable technical advances (28). Ethical concerns surrounding AI in healthcare, particularly regarding data privacy and biased decision-making, may further contribute to delayed adoption in Africa (28). Another chief concern is the absence of data scientists and other AI experts on the continent, leading the majority of African nations to “rely heavily on developed nations for AI technologies to solve critical healthcare challenges” (28). The quality of data produced in Africa and the lack of harmonization thereof could moreover pose a challenge. The quality of the data required to train, test and validate an algorithm determines the accuracy and reliability of the tool, with inconsistent, discrepant data inputs directly equating to erroneous and inaccurate tool outputs. The validation of LEXI with both the SAHPRA and BoMRA data underscored the fact that quality data, adhering to pre-determined guidelines and global best practices, together with supporting data dictionaries and ontologies, are critical for artificial intelligence realization in African medicines regulators.

In view of the above, another important consideration is the ongoing transition toward structured regulatory submissions. The introduction of ICH eCTD v4.0 and the anticipated revision of the CTD Quality guideline [ICH M4Q(R2)] reflect a broader regulatory shift toward standardized, digitally-structured CMC data (29–31). These initiatives aim to improve data interoperability, facilitate regulatory data exchange, and enable more efficient digital review processes. While LEXI was designed to operate within the heterogeneous document environments currently encountered in many African NRAs, it remains adaptable to future structured data ecosystems, with the progressive adoption of structured submissions by African NRAs likely to enhance the performance of not only LEXI, but other AI-assisted regulatory tools. More consistent data structures and standardized terminology would reduce variability in document interpretation and enable more direct extraction of CQAs and other regulatory data elements.

Finally, it is important to assess the readiness of African regulatory authorities to effectively implement AI tools, as this underpins the success of such implementation. Organizational culture (or risk appetite), data availability, technology and, ultimately, strategy all play important roles in shaping and guiding the deployment of AI tools, especially when it impacts human health.

### 4.4 Recommendations

Based on the findings from the above research, the following recommendations should be considered:

1. Tool refinement—LEXI should undergo further independent, blinded external validation within additional jurisdictions, particularly in other African ML3 NRAs, by a larger assessor cohort to strengthen generalizability and allow refinement of the tool for eventual broader African and global regulatory use. Periodic refinement of the tool on new regulatory data, with assessor-supported validation (including additional performance metrics, such as balanced accuracy, positive and negative predictive values, and cost-weighted measures, to provide a more comprehensive assessment of model performance), could also improve LEXI’s accuracy and support continual model improvement. Future development of LEXI should also incorporate deterministic or rule-based computational modules for mathematically derived criteria, such as API load and therapeutic index, to complement the LLM-based reasoning components and improve overall system robustness.
2. Data and submission standardization—To support the effective implementation of AI-assisted regulatory tools such as LEXI, both African NRAs and applicants should prioritize greater standardization of dossier submissions. NRAs should consider strengthening their dossier submission guidelines in line with global best practices and applicants are encouraged to follow established ICH CTD or eCTD structural conventions, including consistent module organization and standardized file and folder naming practices. A formal ablation study quantifying the impact of dossier variability should be prioritized in future work.
3. Governance, risk, and accountability—As AI tools become more embedded in regulatory functions, NRAs should implement comprehensive governance frameworks outlining human oversight, bias mitigation strategies, traceability requirements, and ethical considerations aligned with emerging global regulatory guidance, such as ISO/IEC 42001 and the NIST AI Risk Management Framework. These frameworks should also incorporate operational controls, including model monitoring (e.g., drift detection), incident response procedures, change management processes, and periodic bias evaluation to ensure ongoing system reliability and accountability.
4. In-house Expertise—African NRAs are encouraged to enhance the capabilities of their personnel by providing foundational training in artificial intelligence, fostering both understanding and acceptance of the technology. Additionally, agencies should consider actively recruiting data scientists and AI specialists to support and lead the integration of AI tools within their organizations.

## 5 Limitations

Although this study demonstrated the feasibility and strong predictive performance of LEXI, one of the limitations may be that it has only been validated in two African NRAs, and that further validation in other NRA environs could have produced a more robust tool, revealing additional performance considerations that were not identified in this study. However, as the tool is at present at a proof-of-concept stage, there will be further opportunity to iterate and validate LEXI in different settings reflective of other product dosage forms, dossier requirements and assessment practices. A second limitation is that the tool’s predictive performance is highly dependent on the quality, consistency, and structure of the input data and, as such, successful algorithmic outputs will be hindered by divergent and substandard data inputs, when deployed within other NRAs. A further limitation is that formal inter-rater reliability between assessors was not quantified (e.g., using intraclass correlation coefficients or Cohen’s kappa), which may be considered in future studies to further characterize assessor variability. Another limitation is that the system does not currently generate probabilistic outputs, precluding formal calibration analysis; future iterations may incorporate confidence scoring to support this. In addition, the use of an overall accuracy threshold as the primary acceptance criterion does not fully account for the asymmetric risk associated with misclassification, and future validation studies should consider sensitivity-based thresholds prioritizing detection of high-risk cases.

## 6 Conclusion

LEXI represents a proof-of-concept for the secure, transparent, and contextually relevant use of generative AI in pharmaceutical regulation. By combining large language modeling, retrieval-augmented reasoning, as well as rigorous validation within a locally controlled environment, LEXI demonstrates how AI can enhance the efficiency, reproducibility, and transparency of regulatory assessments while at the same time adhering to data governance and ethical requirements.

The validation of LEXI across two African regulatory authorities, namely SAHPRA and BoMRA, illustrates the feasibility of AI-assisted risk-based assessments within resource-constrained settings. This approach shortens review timelines through consistent, evidence-based risk allocations to generic medicines, suggesting its potential utility in supporting the management of application backlogs in LMICs, subject to further validation.

Data protection and safety remain critical prerequisites for AI adoption in regulatory systems, particularly within LMIC settings. Whether these tools are hosted locally within the NRAs or on a secure cloud, supported by encryption, access controls, and comprehensive auditability, it is important that AI-driven regulatory tools be implemented without jeopardizing confidentiality, thereby fostering institutional trust and supporting wider digital transformation across African NRAs.

Although continuous human oversight remains essential to safeguard the accuracy and accountability of algorithmic decision-making, iterative learning from successive pilot implementations will enable LEXI to evolve into a more robust, domain-specific regulatory tool. With structured feedback loops and standardized data inputs, the system could serve as a digital public good, potentially adaptable to other NRAs seeking to reduce the delays in providing generic medicines at affordable cost to disadvantaged communities.

The integration of AI into regulatory workflows marks a transformative step toward smarter, data-driven health governance. To sustain this momentum, future efforts should focus on expanding LEXI’s knowledge base, incorporating advanced reasoning capabilities, and establishing governance frameworks that ensure transparency, explainability, and long-term sustainability.

## Figures and Tables

**Table:** 

Term	Definition/Description
Artificial intelligence (AI)	Simulation of human intelligence by machines to perform tasks such as reasoning, learning, and decision-making; used to automate regulatory assessments.
Audit trail	Chronological record of system activities ensuring traceability and accountability of data processing and decisions.
BAAI/bge-base-en-v1.5 embedding model	This model converts English text into dense vector embeddings, facilitating tasks like semantic similarity search and information retrieval.
Backend/frontend	Backend manages data and processing logic; frontend is the user-facing interface for document upload and review.
Confusion matrix	A confusion matrix, also known as an error matrix, is a specific table layout that allows visualization of the performance of an algorithm. The confusion matrix helps assess classification model performance in machine learning by comparing predicted values against actual values for a dataset.
Critical quality attributes (CQAs)	Key physical, chemical, or biological properties that ensure product quality, such as purity, potency, and dissolution rate.
Data sovereignty	Principle that data remains under the jurisdiction of the country where it is collected or processed.
Docker	Containerization platform ensuring software reproducibility and secure deployment across computing environments.
Electronic common technical document (eCTD)	Standardized electronic format for submitting pharmaceutical dossiers to regulatory authorities.
Encryption	Process of encoding information to protect it from unauthorized access and maintain confidentiality.
Good automated manufacturing practice (GAMP5)	International guideline for validating and maintaining computerized systems used in regulated industries.
Installation qualification (IQ)	Verification that software is installed and configured according to specifications.
Large language model (LLM)	AI model trained on vast text datasets to understand and generate human-like language; e.g., Llama 3.1 used in LEXI.
Local hosting	Running systems on internal servers to ensure data sovereignty and compliance with national data protection laws.
Machine learning (ML)	A subset of AI enabling systems to learn from data and improve over time without explicit programming.
Natural language processing (NLP)	AI field focused on enabling computers to read, interpret, and generate human language; applied in document analysis and summarization.
OCRMyPDF	OCRmyPDF is a Python application and library that adds text “layers” to images in PDFs, making scanned image PDFs searchable.
Ollama framework	Local deployment environment for large language models, maintaining security within the host organization’s infrastructure.
Operational qualification (OQ)	Testing to confirm that software functions as designed in the intended environment.
Optical character recognition (OCR)	Technology that converts scanned or image-based documents into machine-readable text.
PDFPlumber	PDFPlumber is a Python tool for extracting text, tables, images, and layout details from PDFs with high accuracy.
Performance qualification (PQ)	Validation confirming system performance under actual operating conditions (User Acceptance Testing).
PyMuPDF	PyMuPDF is a high-performance Python library for data extraction, analysis, conversion & manipulation of PDF (and other) documents.
PyPDF2	PyPDF2 is a free and open-source pure-python PDF library capable of splitting, merging, cropping, and transforming the pages of PDF files.
Retrieval-augmented generation (RAG)	Combines language models with a structured knowledge base to generate grounded, context-aware outputs.
Sensitivity (true positive rate)	Sensitivity is the proportion of actual positives that are correctly identified. A high sensitivity equates to few false negatives.
Source code escrow	Source code escrow is a legal arrangement where a software vendor deposits their source code with a neutral third-party agent to protect the user if the vendor goes bankrupt, stops supporting the software, or breaches contract, allowing the user to access the code and maintain business continuity.
Specificity (true negative rate)	Specificity is the proportion of actual negatives that are correctly identified. A high specificity equates to few false positives.
Technology stack	The set of programming languages, frameworks, and tools used to develop an application; LEXI used Python, Docker, and LlamaIndex.
Vector database	Specialized database that stores information as numerical vectors, enabling semantic search by meaning rather than keywords.

## Data Availability

Due to the confidential nature of regulatory product dossiers, raw data cannot be publicly shared. De-identified and aggregated data supporting the conclusions of this study, as well as selected extracts from risk assessment outputs with identifying information removed, will be made available (subject to applicable data-sharing agreements and NRA approvals) by the authors upon reasonable request.
