# Supplementary material for: Development and validation of an artificial intelligence proof-of-concept tool for risk-based quality assessment of generic medicines: a South African case study
Source: Front Med (Lausanne). 2026 May 27;13:1811333. doi: 10.3389/fmed.2026.1811333 (PMC13250854; doi:10.3389/fmed.2026.1811333)
Supplement: Supplementary file 1 [file Table_1.pdf]

Supplementary Material

Table S1: The evolution of the LEXI technology stack

**Tech Stack Components**	**LEXI Tech Stack**
Hardware: The Physical Foundation	LEXI operates on local high-performance servers located within the NRA’s secure infrastructure (but can also be deployed to the cloud).
Operating System: The Platform for All Processes	The system runs on a Linux-based operating system, known for its stability, flexibility, and strong security record.
Database System: LEXI’s Memory and Knowledge Base	LEXI relies on a combination of databases to store and organize regulatory information.<br><br>These databases hold both structured data (like tables of drug names or registration numbers) and unstructured data (like PDF reports and scanned documents).<br><br>A vector database, powered by the LlamaIndex and BAAI/bge-base-en-v1.5 embedding model, allows the system to “understand” and search documents by meaning, not just by keywords.
Backend: The Engine Room	LEXI’s backend is written in Python 3.9, which is a programming language widely used in scientific computing and AI.<br><br>It includes specialized software packages:<br><br>• LlamaIndex (to manage document search and retrieval)<br><br>• OCRMyPDF, PyPDF2, and pdfplumber (to extract text from PDFs and scanned images)<br><br>• Selenium (to automatically gather data from regulatory websites)
Frontend: The User Interface	It’s a web-based dashboard that allows users to upload documents, submit queries, and view AI-generated risk assessments.

Networking: The Secure Connections	LEXI operates within a closed network - meaning it does not rely on the public internet for any of its processing and no confidential data ever leaves the secure environment of the NRA.<br><br>• Internal connections between the AI engine, database, and user interface are encrypted, ensuring that no unauthorized access is possible.<br><br>• When LEXI needs to gather information from trusted external sources (like the WHO Prequalification database or the EDQM listings), it uses controlled and automated web scraping through Selenium, under strict security permissions.
Cloud and Infrastructure Services: Containerization and Deployment	LEXI is deployed using Docker containers, which act like sealed digital “boxes” that contain everything the system needs to run - the software, libraries, and configurations.<br><br>Unlike many modern AI tools, LEXI does not rely on commercial cloud services such as Amazon Web Services or Microsoft Azure.<br><br>Instead, it is hosted entirely within the NRA’s internal servers, ensuring data sovereignty and compliance with local data protection laws.
