# Supplementary material for: Development and validation of an artificial intelligence proof-of-concept tool for risk-based quality assessment of generic medicines: a South African case study
Source: Front Med (Lausanne). 2026 May 27;13:1811333. doi: 10.3389/fmed.2026.1811333 (PMC13250854; doi:10.3389/fmed.2026.1811333)
Supplement: Supplementary file 2 [file Table_2.pdf]

frontiers

Supplementary Material

Table S2: Model Card–Style Description of the LEXI AI System

Category	Description
**Model Type**	Retrieval-Augmented Generation (RAG) system combining a large language model with a structured regulatory knowledge base
**Primary Use Case**	Automated risk-based assessment (RBA) and triage of Chemistry, Manufacturing and Controls (CMC) data in generic medicine dossiers
**Intended Users**	National regulatory authority (NRA) assessors and regulatory scientists
**Development Context**	Designed for use in low- and middle-income country (LMIC) regulatory environments with heterogeneous dossier formats
**Training Data (System Calibration)**	210 SAHPRA generic medicine dossiers from the Backlog Clearance Project (BCP)
**Data Split (Internal Validation)**	Training/validation set: 157 dossiers (75%); Internal test set: 53 dossiers (25%); split performed randomly
**External Validation Data**	30 SAHPRA dossiers (not part of BCP); 30 BoMRA dossiers
**Input Data Types**	PDF dossiers (including scanned documents), CTD Modules (primarily Module 3), regulatory reports, and structured external reference data
**Preprocessing and Document Handling**	Optical Character Recognition (OCR) using OCRMyPDF; PDF parsing using PyPDF2, pdfplumber, and PyMuPDF; document classification and section identification workflows applied to heterogeneous dossier formats
**Embedding Model**	BAAI/bge-base-en-v1.5
**Large Language Model (LLM)**	Llama 3.1 (8B parameters), deployed locally via Ollama; no fine-tuning performed
**RAG Framework**	LlamaIndex for document indexing, retrieval, and context augmentation
**Context Window**	Up to 128,000 tokens
**External Data Integration**	European Directorate for the Quality of Medicines (EDQM) CEP database; WHO Prequalification Programme; DrugBank
**Output Type**	Structured risk classification, criterion-level scoring, and traceable summaries of critical quality attributes (CQAs)
**Performance Metrics**	Overall predictive accuracy: 91.7%; Sensitivity: 97%; Specificity: 86% (external validation, n=60 dossiers)
**Human Oversight**	Human-in-the-loop validation; final regulatory decisions remain with expert assessors
**Explainability and Traceability**	Outputs linked to source documents and retrieval context; audit trails maintained for all processing steps

Category	Description
**Limitations**	Performance dependent on data quality, document structure, and consistency; variability observed with non-standard dossier formats (e.g., BoMRA submissions)
**Ethical and Governance Considerations**	Designed for secure, local deployment to ensure data sovereignty; alignment with emerging AI governance frameworks (e.g., ISO/IEC 42001, NIST AI RMF) planned as part of future work
**Adaptability**	Modular architecture allows integration of improved LLMs, embedding models, and structured regulatory data (e.g., future eCTD v4.0 environments)
**Versioning Note**	Model and embedding choices reflect state-of-the-art at time of development; rapid advancements in AI may lead to improved performance in future implementations
