# Supplementary material for: Development and validation of an artificial intelligence proof-of-concept tool for risk-based quality assessment of generic medicines: a South African case study
Source: Front Med (Lausanne). 2026 May 27;13:1811333. doi: 10.3389/fmed.2026.1811333 (PMC13250854; doi:10.3389/fmed.2026.1811333)
Supplement: Supplementary file 3 [file Table_3.pdf]

Supplementary Material

Table S3: Error Log containing 20 PQ tests after initial SAHPRA external validation

**Error Log**		
Test ID	**Error Description**	**Technical Notes**
URS033	Expected accuracy on overall criteria score and final output 95%. Actual accuracy 63%. Accuracy below expected accuracy.	The accuracy of the high/low classification for the scoring of this criterion is 100%, indicating that the incorrect score did not have any impact on the final output.
URS034	Expected accuracy on overall final output 95%. Actual accuracy 63%. Accuracy below expected accuracy.	The accuracy of the high/low classification for the scoring of this criterion is 100%, indicating that the incorrect score did not have any impact on the final output.
URS035	Expected accuracy on overall criteria score 95%. Actual accuracy 63%. Accuracy below expected accuracy.	The accuracy of the high/low classification for the scoring of this criterion is 100%, indicating that the incorrect score did not have any impact on the final output.
URS036	Expected accuracy on RA1 score and final 95%. Actual accuracy 39%. Accuracy below expected accuracy.	The accuracy of the high/low classification for the scoring of this criterion is 100%, indicating that the incorrect score did not have any impact on the final output.
URS037	Expected accuracy on RA2 score and final 95%. Actual accuracy 40%. Accuracy below expected accuracy.	The accuracy of the high/low classification for the scoring of this criterion is 76%, indicating that the incorrect score did have an impact on the final output.
URS038	Expected accuracy on RF1 score and final 95%. Actual accuracy 3%. Accuracy below expected accuracy.	The accuracy of the high/low classification for the scoring of this criterion is 100%, indicating that the incorrect score did not have any impact on the final output.

URS039	Expected accuracy on RF2 score and final 95%. Actual accuracy 10%. Accuracy below expected accuracy.	The accuracy of the high/low classification for the scoring of this criterion is 37%, indicating that the incorrect score did not have any impact on the final output.
URS040	Expected accuracy on RB1 score and final 95%. Actual accuracy 70%. Accuracy below expected accuracy.	The accuracy of the high/low classification for the scoring of this criterion is 100%, indicating that the incorrect score did have an impact on the final output.
URS041	Expected accuracy on A1 score and final 95%. Actual accuracy 52%. Accuracy below expected accuracy.	The accuracy of the high/low classification for the scoring of this criterion is 98%, indicating that the incorrect score did not have any impact on the final output.
URS042	Expected accuracy on A2 score and final 95%. Actual accuracy 77%. Accuracy below expected accuracy.	The accuracy of the high/low classification for the scoring of this criterion is 100%, indicating that the incorrect score did not have any impact on the final output.
URS043	Expected accuracy on A3 score and final 95%. Actual accuracy 80%. Accuracy below expected accuracy.	The accuracy of the high/low classification for the scoring of this criterion is 100%, indicating that the incorrect score did not have any impact on the final output.
URS044	Expected accuracy on A4 score and final 95%. Actual accuracy 81%. Accuracy below expected accuracy.	The accuracy of the high/low classification for the scoring of this criterion is 100%, indicating that the incorrect score did not have any impact on the final output.
URS045	Expected accuracy on A5 score and final 95%. Actual accuracy 35%. Accuracy below expected accuracy.	The accuracy of the high/low classification for the scoring of this criterion is 94%, indicating that the incorrect score did have an impact on the final output.
URS046	Expected accuracy on A6 score and final 95%. Actual accuracy 81%. Accuracy below expected accuracy.	The accuracy of the high/low classification for the scoring of this criterion is 78%, indicating that the incorrect score did have an impact on the final output.

URS047	Expected accuracy on F1 score and final 95%. Actual accuracy 70%. Accuracy below expected accuracy.	The accuracy of the high/low classification for the scoring of this criterion is 80%, indicating that the incorrect score did have an impact on the final output.
URS048	Expected accuracy on F2 score and final 95%. Actual accuracy 73%. Accuracy below expected accuracy.	The accuracy of the high/low classification for the scoring of this criterion is 70%, indicating that the incorrect score did have an impact on the final output.
URS049	Expected accuracy on F3 score and final 95%. Actual accuracy 87%. Accuracy below expected accuracy.	The accuracy of the high/low classification for the scoring of this criterion is 100%, indicating that the incorrect score did not have any impact on the final output.
URS050	Expected accuracy on F4 score and final 95%. Actual accuracy 93%. Accuracy below expected accuracy.	The accuracy of the high/low classification for the scoring of this criterion is 97%, indicating that the incorrect score did not have any impact on the final output.
URS051	Expected accuracy on F5 score and final 95%. Actual accuracy 93%. Accuracy below expected accuracy.	The accuracy of the high/low classification for the scoring of this criterion is 97%, indicating that the incorrect score did not have any impact on the final output.
URS052	Expected accuracy on B1 score and final 95%. Actual accuracy 93%. Accuracy below expected accuracy.	The accuracy of the high/low classification for the scoring of this criterion is 97%, indicating that the incorrect score did not have any impact on the final output.

frontiers
